# The stored product beetles *Lasioderma serricorne* and *Stegobium paniceum* are associated with a flexible and hidden diversity of *Symbiotaphrina* symbionts

**DOI:** 10.1038/s41598-025-34676-y

**Published:** 2026-01-08

**Authors:** Nick Alina, Schellenberg René, Athanassiou Christos G., Adler Cornel, Engl Tobias

**Affiliations:** 1 Evolutionary Ecology, iomE, Johannes Gutenberg-University Mainz, Mainz, Germany; 2 Department of Insect Symbiosis, Max-Planck Institute for Chemical Ecology, Jena, Germany; 3 Laboratory of Entomology and Agricultural Zoology, Department of Agriculture, Crop Production and Rural Environment, University of Thessaly, Thessaly, Greece; 4 Stored Crop Protection, Federal Research Centre for Cultivated Plants, Berlin, Germany

**Keywords:** Anobiidae, Symbiosis, Symbiotaphrina, Yeast-like symbiont, Stored-product insects, Ecology, Ecology, Evolution, Microbiology, Molecular biology, Zoology

## Abstract

The stored product pests *Lasioderma serricorne* and *Stegobium paniceum* (Coleoptera: Ptinidae: Anobiinae) have been described to harbour *Symbiotaphrina kochii* and *Symbiotaphrina buchneri* yeast-like symbionts (YLS) respectively, based on axenic cultivation from symbiotic organs. While the nutritional benefits provided by the YLS are well characterized, molecular studies focusing on variability or stability in the systems are lacking. Here we address that research gap by investigating the diversity and stability of Anobiid – *Symbiotaphrina* symbiosis in multiple populations. Amplicon sequencing of the fungal internal transcribed spacer (ITS) from collected and lab-reared populations revealed that populations from different origins were associated with similar YLS strains. These associations remained stable during rearing. However, only one *L. serricorne* population was associated with *Sy. kochii*, the others were associated with *Sy. buchneri.* Most *St. paniceum* samples were associated with a *Symbiotaphrina* species that could neither be identified as *Sy. buchneri*, nor *Sy. kochii.* Cultivation and subsequent phylogenetic analysis of the partial rRNA operon of YLS revealed a novel *Symbiotaphrina* clade. Our results indicate more flexible associations than previously assumed, however only with members of the genus *Symbiotaphrina*. The ecological relevance needs further analyses, but highly variable in vitro growth could indicate extensive differences in YLS capabilities.

**Supplementary Information:**

The online version contains supplementary material available at 10.1038/s41598-025-34676-y.

## Introduction

Microbes are omnipresent in the environment: they surround, impact and interact with every organism forming diverse associations, communities, and ecosystems^1^. The highly diverse associations between insects and microbes can be major drivers of evolution^2^. Besides antagonistic microbes including competitors, parasites and pathogens, mutualistic microbial symbionts contribute often crucially to their host’s metabolism enabling the insects to adapt to new ecological niches^3^. The supplementation of essential amino acids, vitamins or sterols by microbial mutualists enables various insects to feed on nutrient poor or unbalanced diets^4,5^. Many nutritional mutualisms are highly stable and conserved across taxonomic groups while defensive or detoxifying ones can be more flexible and thereby exhibit higher variation within the microbial partners^4,6^. Symbiont localization might also influence the stability of a symbiosis. Endosymbionts living inside the host’s body are isolated from the environment while ectosymbionts living outside of the host’s body cavity (including the gut) encounter other microbes more frequently^7^. Accordingly, exchange of microbes and/or genetic material encoding metabolic functions can occur more frequently for these ectosymbionts.

Symbiont harbouring tissues can have various degrees of complexity ranging from gut invaginations or crypts to distinct organs called bacteriomes or mycetomes (depending on whether they house bacteria or fungi) where microbes can occur intra- and/or extracellularly^6^. Examples of both types of symbiont harbouring organs can be found in anobiid beetles (Coleoptera: Ptinidae: Anobiinae, also known as Anobiidae) which are associated with yeast-like symbionts (YLS)^8–10^. Larvae and adults harbour the YLS intracellularly in specialized epithelial cells (mycetocytes) located in evaginations of the midgut (mycetomes)^11^. Additionally, female adults harbour extracellular YLS in intersegmental tubules that are connected to the reproductive organs^11^. During oviposition, the egg surface is smeared with YLS, the newly hatched larvae consume a part of the eggshell and YLS can colonise the mycetocytes^8,11^. In contrast to most other symbioses, these dual microbe harbouring organs represent an intermediate stage between strictly extracellular and intracellular symbioses while the intermittent intracellular life stage is likely a strong selection pressure for specific microbes capable of invading but not overexploiting this host environment.

Although, the presence of YLS has been described in many anobiids^8,12^ cultivation and characterization were only successful from five anobiid species^13^. Out of these five species, only the symbiosis of the tabaco beetle *Lasioderma serricorne* and the drugstore beetle *Stegobium paniceum* with their YLS are relatively well studied. *L. serricorne* was described to be associated with *Symbiotaphrina kochii*^14^ and *St. paniceum* was described to be associated with *Symbiotaphrina buchneri*^15^ (Ascomycota: Symbiotaphrinales: Symbiotaphrinaceae^16^. Remarkably, neither *L. serricorne* nor *St. paniceum* feed on (dead) wood like other anobiids but have diverged to be stored product pests. Both beetles feed on a variety of stored products, mainly dead plant material, making them economically important pests^17,18^.

Since the transmission of YLS happens via the egg surface, egg surface sterilization can yield symbiont-free (aposymbiotic) beetles. Koch^19^ described that aposymbiotic larvae developed slower than symbiotic control larvae and eventually died before metamorphosis. Further experiments with symbiotic and aposymbiotic insects inferred that *Sy. kochii* and *Sy. buchneri* supplement *L. serricorne* and *St. paniceum* with B -vitamins^12,20^, sterols^21,22^ and amino acids^23,24^ as the addition of those compounds to the diet could rescue aposymbiotic larvae. Although *L. serricorne* and *St. paniceum* benefit greatly from the nutrients provided by their respective *Symbiotaphrina* YLS, the transmission route via the egg surface with exposure to the environment suggests a certain flexibility in these systems. Interestingly, aposymbiotic insects can also acquire YLS from the environment, for example from faeces of conspecifics^21,25^. Reinfection experiments with *L. serricorne* and *St. paniceum* and *Sy. kochii* and *Sy. buchneri* showed that the symbionts are exchangeable between the beetles with no or little difference in their development^25^. Similarly, experimental infections with a related yeast-like fungus (*Cyberlindnera jadinii*, formerly *Torulopsis utilis)* established intracellularly, however with a strongly exaggerated, seemingly pathogenic infection spread into gut epithelial tissues beyond the mycetome^26^. Despite the specific requirements for the intracellular life stage this system seems to be able to maintain a certain flexibility regarding the microbial partner. However, the diversity of beetle and YLS association has only been studied by culture dependent methods and without a focus on variability in the infecting species or strains of YLS. The necessity for more sensitive approaches, e.g. offered by molecular methods is even more imperative, since Baral et al.^16^ found *Symbiotaphrina* species closely related to *Sy. buchneri* and *Sy. kochii* on decaying wood – an ecological niche shared with many anobiid beetles. They suggest that *Sy. buchneri* and *Sy. kochii* have an undiscovered free- living, sexual morph and that the newfound species might have an undiscovered symbiotic relationship with arthropods.

The existence of a free-living state of symbionts has multiple implications on selective pressures e.g. retention of non-mutualistic capabilities, but also on the stability and consistency of the associations found in beetles. A regular exchange with non-beetle associated populations should result in higher variability, possibly also higher flexibility in the beetle associated YLS strains or species. This study aims to investigate the diversity and stability of the symbiosis between *L. serricorne/St. paniceum* and yeast-like symbionts between and within multiple populations in Germany. The fungal community of whole beetles was analysed in different populations collected from various sources and in subsequent generations in laboratory cultures. This allowed the comparison of the fungal community in between populations but also in between generations of single populations, giving insight into the diversity of the community, as well as its stability during lab rearing. In a further approach to resolve the putative *Symbiotaphrina* strains better, the major YLS of two populations of *L. serricorne* and two populations of *St. paniceum* were isolated and phylogenetically classified based on longer amplicons spanning the rRNA operon.

## Results

### Fungal community analysis

The fungal communities of multiple anobiid populations were analysed using ITS MiSeq amplicon sequencing. For the populations that were newly established in the lab, *L. serricorne* ‘chickpea’, *L. serricorne* ‘greece’, *St. paniceum* ‘chili’ and *St. paniceum* ‘cornflakes’, two generations were considered to detect possible changes in the community during lab rearing (“F0” before lab rearing, “Fx” after lab rearing). Furthermore, the identified and isolated *Symbiotaphrina* strains were phylogenetically classified.

**Fig. 1:**
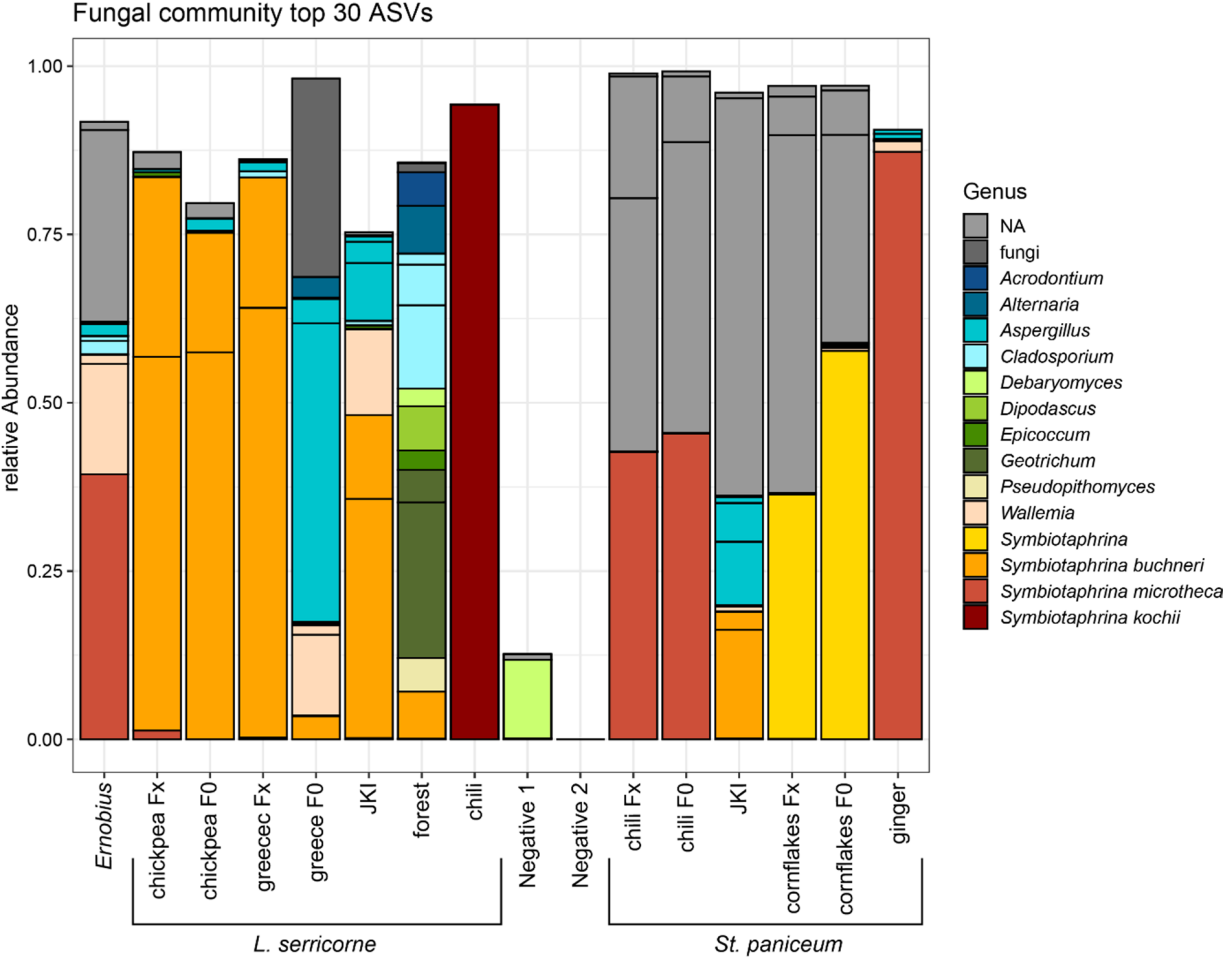
Composition and relative abundance of fungal genera in anobiid beetles. Amplicon Sequence Variants (ASVs) were inferred from merged forward and reverse reads of ITS Illumina paired end sequencing, top 30 ASVs named, remaining summarized as “other”; undetermined fungal ASVs are summarised as fungi. ASV_1, ASV_3 & ASV_21 could not be assigned to fungi and are labelled as NA; JKI = Julius Kühn Institute, F0 = native beetles (“generation 0”), Fx = lab reared beetles (“generation x”); negative 1 = negative control run 1; negative 2 = negative control run 2.

The fungal community was more diverse in wild anobiids compared to lab-reared beetles. The fungal communities of lab-reared populations were mostly stable over time (Fig. 1). Only in *L. serricorne* ‘greece’ the relative abundances changed upon establishment in the lab: the relative abundance of environmental fungi reduced and the relative abundance of symbiotic *Symbiotaphrina* increased.

*Symbiotaphrina* could be identified on genus level in all populations of *L. serricorne* and *St. paniceum* as well as in *Ernobius*. *Symbiotaphrina buchneri* could be identified throughout the different lab generations of *L. serricorne* ‘chickpea’ and ‘greece’, *L. serricorne* ‘JKI’, *L. serricorne* ‘forest’ and in *St. paniceum* ‘JKI’, while *Sy. kochii* was only found in *L. serricorne* ‘chili’. The *Symbiotaphrina* species in *St. paniceum* ‘chili’, *St. paniceum* ‘ginger’ and *Ernobius* was identified as *Sy. lignicola* based on the fungal community analysis. However further data base comparison revealed higher identity with *Sy. microtheca*. We could not assign the *Symbiotaphrina* species in *St. paniceum* ‘cornflakes’ to *Sy. buchneri* nor *Sy. kochii* based on the fungal community analysis alone. We therefore refer to this as “novel *Symbiotaphrina*” strain. The sequence from this “novel *Symbiotaphrina*” ASV (ASV 4) was later compared to sequences of isolated *Symbiotaphrina* strains obtained by sanger sequencing to resolve its phylogenetic placement (see below ‘Phylogenetic placement of *Symbiotaphrina* isolates & YLS of wild anobiids’). Several fungal ASVs that could not be identified further were highly abundant in *St. paniceum* samples and in *Ernobius*.

**Fig. 2:**
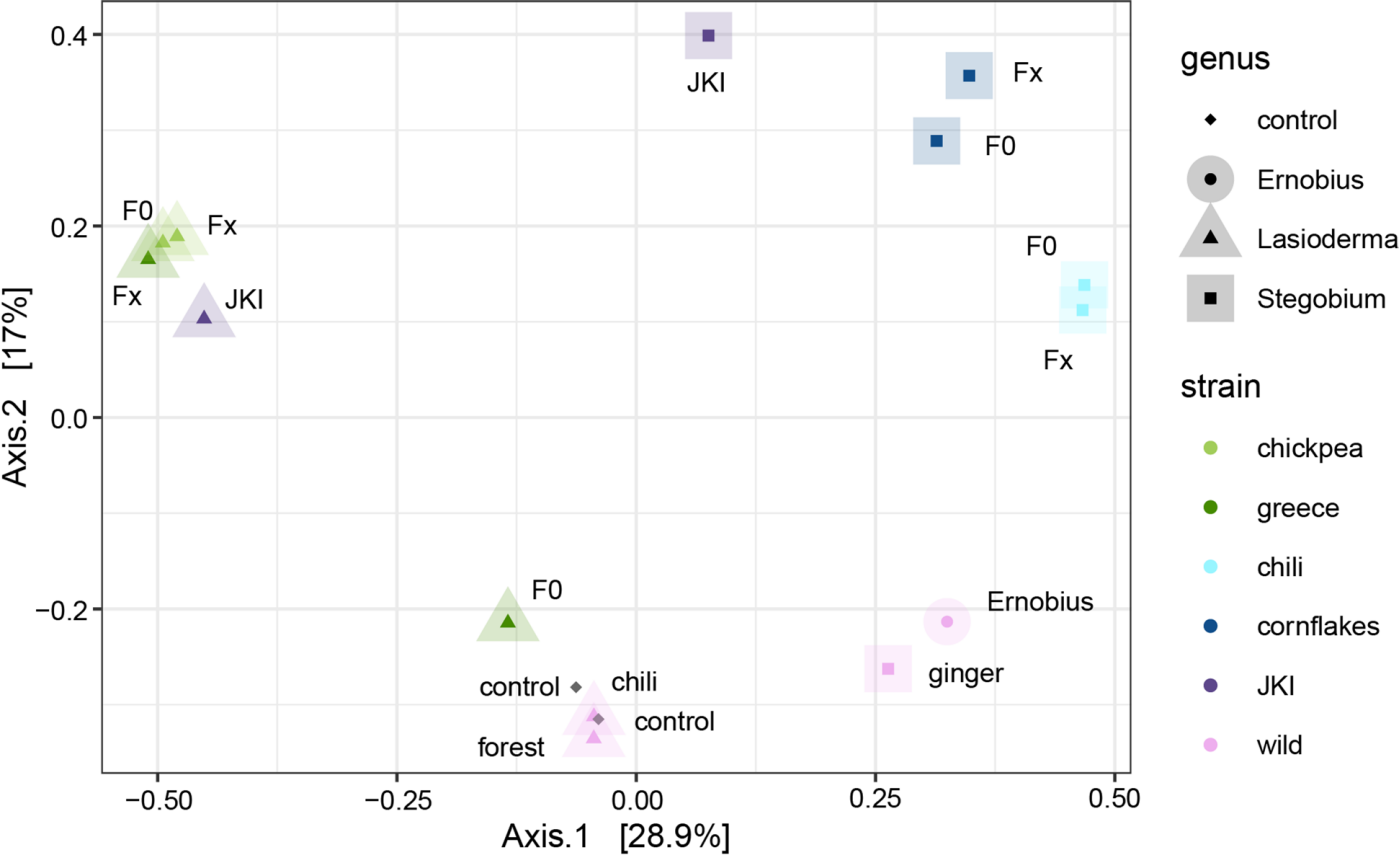
Principal Coordinate Analysis of fungal communities from anobiid beetles based on Bray-Curtis dissimilarity.

The principal coordinate analysis (Fig. 2) showed high similarity between samples of the same genus for both *L. serricorne* and *St. paniceum*. The fungal communities were clearly separated by host species, which is supported by permutational multivariate analysis of variance (PERMANOVA,df = 3, *R*^*2*^ = 0.36, *p* = 0.002). *L. serricorne* ‘chickpea’, *St. paniceum* ‘chili’ and *St. paniceum* ‘cornflakes’ were similar within a generation but distinct between populations. Only the fungal community of *L. serricorne* ‘greece’ had changed during lab rearing, being very close to *L. serricorne* ‘chickpea’ after rearing.

### Phylogenetic placement of *Symbiotaphrina* isolates & YLS of wild anobiids

**Fig. 3:**
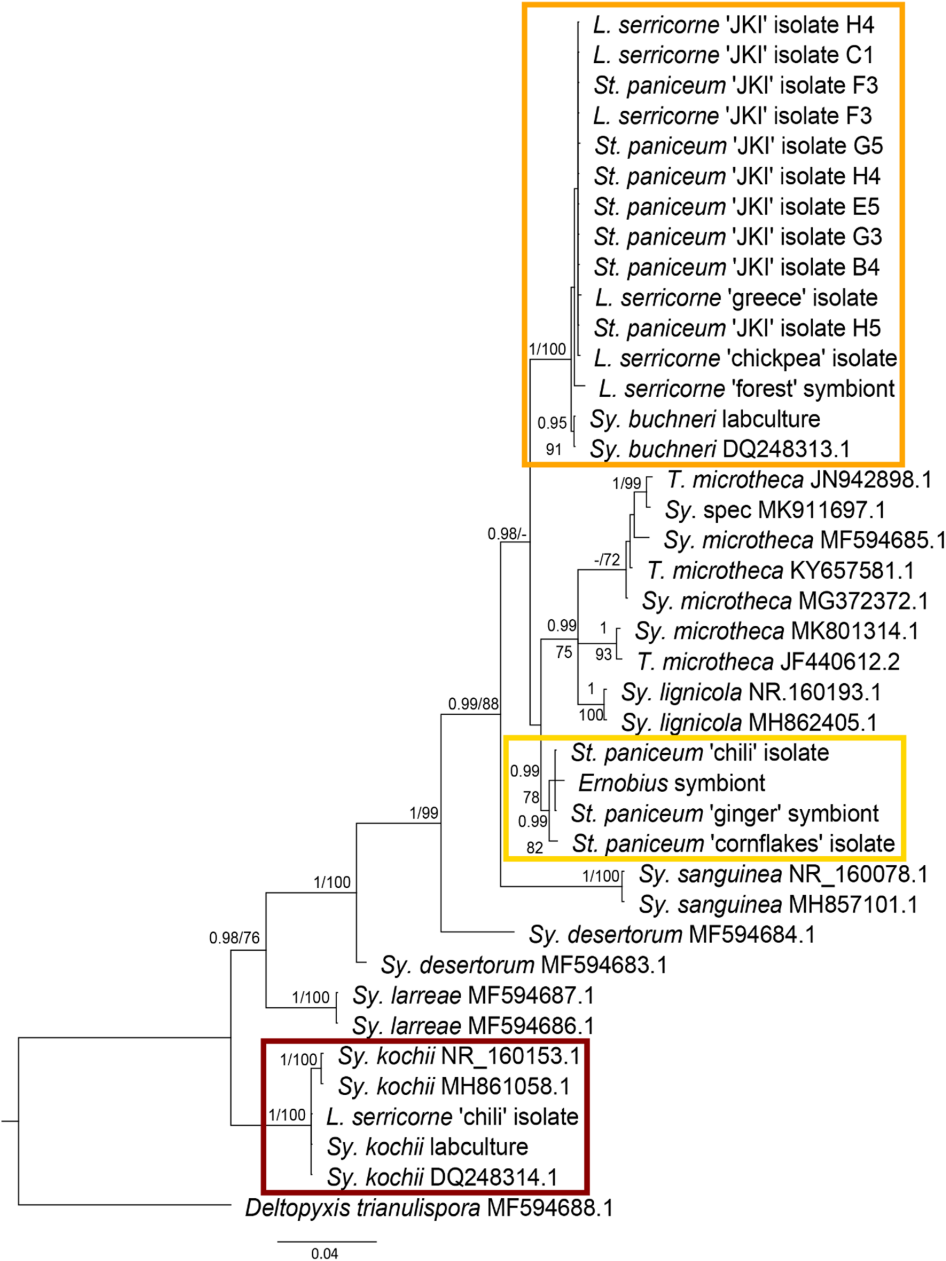
Phylogenetic placement of YLS isolates and uncultured anobiid symbionts. Combined gene tree of MrBayes & RAxML phylogenies (individual trees in Supplementary Figs. 2 + 3). Posterior probabilities (left) & bootstrap support values (right) are given on branches, values with weak support (posterior probabilities < 0,9, bootstraps < 70) are not shown. Orange box: *Sy. buchneri* clade; yellow box: novel *Symbiotaphrina* clade; red box: *Sy. kochii* clade.

Axenic cultivation attempts yielded colonies from most examined species with colony and cell phenotypes similar to *Sy. buchneri* and *kochii*, although with slight deviation in colony coloration ranging from pale white to light beige as well as cell morphology (Supplementary Fig. 1). *Sy. buchneri* and *kochii* displayed almost perfectly spherical cells, with only infrequent elongated variations, the isolates clustering with *Sy. buchneri* cell were round to slightly oval, the novel *Symbiotaphrina* clade isolates oval (up to twice as long as wide), whereas the *Sy. kochii* clade isolate exhibited a pronounced conical cell morphology. The partial rRNA operons of all *Symbiotaphrina* strains isolated in axenic cultures (Table 2) and from the *Ernobius*, *L. serricorne* ‘forest’ and *St. paniceum* ‘ginger’ specimen were amplified from DNA extracts, purified, sanger sequenced and analysed to complete a phylogenetic classification of *Symbiotaphrina* isolates. The resulting trees from Maximum Likelihood and Bayesian analysis showed the same topology and were thus combined (Fig. 3; individual, unedited trees Supplementary Figs. 2 + 3).

All *Stegobium* ‘JKI’ isolates and *Lasioderma* ‘JKI’ isolates clustered together with the isolates from *Lasioderma* ‘greece’ and *Lasioderma* ‘chickpea’ as well as the *L. serricorne* ‘forest’ symbiont. They formed a sister clade to sequences of the *Sy. buchneri* type strain NBRC10845 cultivated in the lab and those previously deposited in databases (pp:1; bs: 100; Fig. 3: orange box: “*Sy. buchneri* clade”). The *Stegobium* ‘chili’ and *Stegobium* ‘cornflakes’ isolate clustered together with the *Ernobius* and *St. paniceum* ‘ginger’ symbionts in a sister clade of the *Sy./T. microtheca* sequences (pp: 99; bs: 82; Fig. 3 yellow box: “novel *Symbiotaphrina* clade”). The *Lasioderma* ‘chili’ isolate and lab culture of *Sy. kochii* CBS250.77 clustered with deposited *Sy. kochii* sequences (pp: 1; bs: 100; Fig. 3: red box: “*Sy. kochii* clade”).

**Fig. 4:**
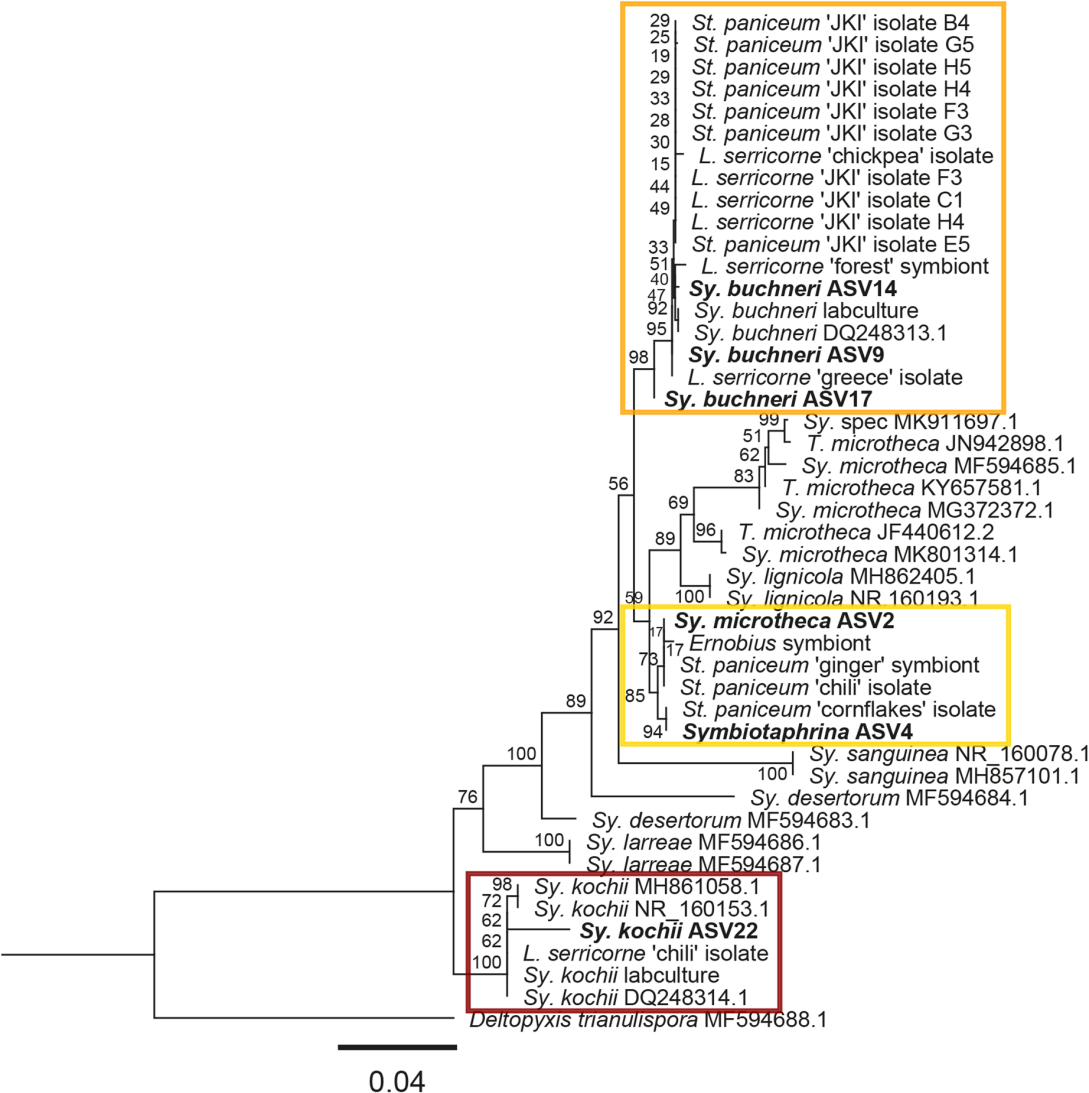
Phylogenetic placement of *Symbiotaphrina* ASVs within YLS phylogeny. Phylogenetic tree based on RAxML phylogeny containing bootstrap support values are given on branches. Orange box: *Sy. buchneri* clade; yellow box: novel *Symbiotaphrina* clade; red box: *Sy. kochii* clade.

To link the phylogenetic placement of *Symbiotaphrina* isolates and symbionts to the results of our community analysis we extracted the *Symbiotaphrina* sequences from the top 30 ASVs (ASV2, ASV4, ASV9, ASV14, ASV17 and ASV22) and run a phylogenetic analysis (RAxML in Geneious, 1000 bootstraps, Fig. 4). ASV9, ASV14 and ASV17 clustered with the “*Sy. buchneri* clade” (orange box) and ASV22 clustered with “*Sy. kochii* clade” (red box) fitting with the previous identification. ASV4 clustered with *St. paniceum* ‘cornflakes’ isolate and ASV2 with *St. paniceum* ‘ginger’ symbiont, *St. paniceum* ‘chili’ isolate and *Ernobius* symbiont, both in the “novel *Symbiotaphrina* clade” (yellow box). ASV2 had been assigned as *Sy. microtheca* in the community analysis but here it is clearly positioned within the “novel *Symbiotaphrina* clade” which shows supported separation from the other *Sy. microtheca* sequences.

### Diagnostic polymerase chain reaction

Based on the phylogenetic position of the *Stegobium* ‘greece’ isolate, *Stegobium* ‘chili’ isolate, *Lasioderma* ‘chickpea’ isolate and *Stegobium* ‘cornflakes’ isolate diagnostic primers for the distinction of the two groups (Fig. 3: orange box buch& yellow box nov) as well as *Sy. kochii* were designed.

The in silico specificity of the new primers was confirmed by counting mismatches: Sym_buch_classic had 100% identity to the sequences of *Sy. buchneri*, *Stegobium* ‘greece’ isolate, *Lasioderma* ‘chickpea’ isolate (17/17 bases) with 4 mismatches to the sequences of *Stegobium* ‘chili’ isolate and *Stegobium* ‘cornflakes’ isolate and seven mismatches to *Sy. kochii* sequence. Sym_novel had 100% identity to the sequences of *Stegobium* ‘chili’ isolate and *Stegobium* ‘cornflakes’ isolate (19/19 bases) with 6 mismatches to the sequences of *Sy. buchneri*, *Stegobium* ‘greece’ isolate, *Lasioderma* ‘chickpea’ isolate and 9 mismatches to *Sy. kochii* sequence.

**Fig. 5:**
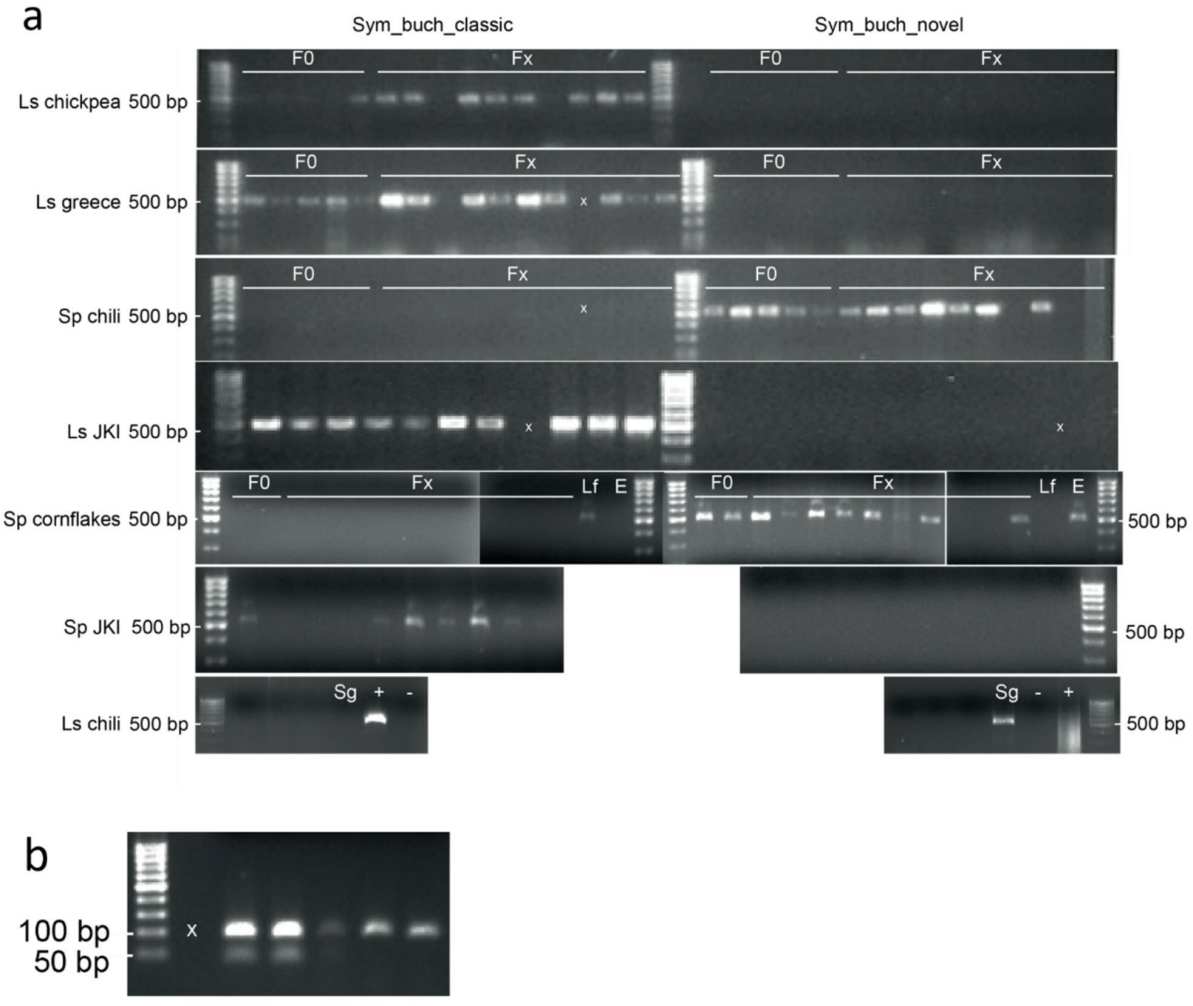
Agarose gel electrophoresis of diagnostic polymerase chain reaction amplicons. (a) Diagnostic primer combinations Sym_buch_classic & Sym_novel are suitable to differentiate *Symbiotaphrina* strains in DNA extracts of whole beetles. Amplification of partial rRNA operon of the *Symbiotaphrina buchneri* strains (ca. 500 bp) from DNA extracts of whole beetles was either successful with Sym_buch_classic or Sym_novel, no sample had PCR success with both primers. PCR using Sym_buch_classic was successful for *L. serricorne* ‘chickpea’ (F0 & Fx), *L. serricorne* ‘greece’ (F0 & Fx), *L. serricorne* ‘JKI’ and *L. serricorne* ‘forest’ (Lf), as well as *St. paniceum* JKI beetle extracts. PCR using Sym_novel was successful for *St. paniceum* ‘chili’ (F0 & Fx), *St. paniceum* ‘cornflakes’ (F0 & Fx), *St. paniceum* ‘ginger’ (Sg) and *Ernobius* sp. (E) beetle extracts. For *L. serricorne* ‘chili’ neither PCR was successful. Ls = *L. serricorne*, Sp = *St. paniceum*, Lf = *L. serricorne* forest, JKI = Julius Kühn Institute, E = *Ernobius*, Sg = *St. paniceum* ginger, F0 = extracts from native beetles (“generation 0”), Fx = lab reared beetles (“generation x”), x = empty slot, +=positive control, - =negative control. Original gels are presented in Supplementary Fig. 4. (b) Diagnostic primers for *Sy. kochii* successfully amplified symbionts of *L. serricorne* ‘chili’. Amplification of partial rRNA operon of the *Symbiotaphrina kochii* strain (ca. 100 bp) from DNA extracts of whole beetles and the YLS isolated from *L. serricorne* ‘chili’; x = empty slot; original gel in Supplementary Fig. 4.

They were applied to all individual samples used in the pooled fungal community analysis (DNA extracts of whole beetles, Fig. 1) to confirm the infection with the different *Symbiotaphrina* strains. For each individual sample, PCR was either successful with primer Sym_buch_classic or with Sym_novel or with neither one (Fig. 5a). No sample had PCR success with both primers. Successful PCRs yielded products of expected 500 bp length. In F0 and Fx generations of *L. serricorne* ‘chickpea’ and ‘greece’, in *L. serricorne* ‘JKI’, *St. paniceum* ‘JKI’ as well as in *L. serricorne* ‘forest’ the occurring *Symbiotaphrina* species could be confirmed to belong to the “*Sy. buchneri”* clade (Fig. 3 orange box, Fig. 1). The *Symbiotaphrina* species in populations of *St. paniceum* ‘chili’ and ‘cornflakes’ as well as *Ernobius* and *St. paniceum* ‘ginger’ belong to the “novel *Symbiotaphrina* isolate” clade (Fig. 3: yellow box, Fig. 1).

A diagnostic PCR using *Sy. kochii* specific primers was successful for *L. serricorne* ‘chili’ beetles and yielded products of ~ 100 bp length (Fig. 5b), confirming the phylogenetic position of their symbiont (Fig. 3: red box).

## Discussion

In this study we investigated the diversity and stability of anobiid – YLS symbiosis, focusing on the interaction between *Lasioderma serricorne* and *Stegobium paniceum* with *Symbiotaphrina* YLS. To account for the complete fungal community in the beetles before and after lab rearing, individuals from each population were pooled and analysed by amplicon sequencing. In addition, the yeast-like symbionts were cultivated and single isolates classified by Sanger sequencing of the rRNA operon. Additionally, we analysed some wild anobiids and *L. serricorne* & *St. paniceum* populations, that were not reared in the lab. We found *Symbiotaphrina* in all populations of *St. paniceum* and *L. serricorne.* While only one *L. serricorne* population was associated with *Sy. kochii*, most carried a strain with a highly similar rRNA operon as *Sy. buchneri*. The freshly collected *St. paniceum* populations all carried a strain that formed a novel cluster in between *Sy. buchneri* and *Sy. microtheca* based on phylogenies of the rRNA operon. While short term cultivation in the lab did not alter the association with the *Symbiotaphrina* species or strain, long term cultures of *L. serricorne* and *St. paniceum* maintained in the same lab were both infected with *Sy. buchneri* strains that could not be differentiated by the rRNA operon sequences. These findings contradict the commonly assumed stable association of *L. serricorne* with *Sy. kochii* and *St. paniceum* with *Sy. buchneri*^10,14,15^, revealing not only flexibility between both so far recognized *Symbiotaphrina* species, but also indicating a potentially novel species.

In the phylogenetic analysis of *Symbiotaphrina* isolates, three clades of *Symbiotaphrina* could be distinguished. *Symbiotaphrina* isolates from both *L. serricorne* ‘JKI’ and *St. paniceum* ‘JKI’ clustered with *Stegobium ‘*greece’ and *Lasioderma* ‘chickpea’ isolates as a sister group to *Sy. buchneri* type strain and lab culture (“*Symbiotaphrina buchneri* clade”, Fig. 3: orange box). *Stegobium* ‘chili’ isolate and *Stegobium* ‘cornflakes’ isolate formed a clade with the *Ernobius* symbiont and *St. paniceum* ‘ginger’ symbiont (“novel *Symbiotaphrina* clade”, yellow box Fig. 3), forming a sister group to the canonical *Sy. buchneri* clade. Only the *L. serricorne* ‘chili’ isolate clustered with *Sy. kochii* (Fig. 3: red box).

The comparison of freshly collected populations upon arrival in the lab (“F0”) and subsequently reared beetles (“Fx”) revealed that beetles harboured stable associations and did not exchange *Symbiotaphrina* species or strains within the observation period of this study. However, the relative abundance changed in some cases, e.g. *Sy. buchneri* abundance was much higher in Fx beetles of *L. serricorne* ‘chickpea’ and ‘greece’ than in F0 beetles. We established the *L. serricorne* ‘chickpea’ lab population from a wild population, while *L. serricorne* ‘greece’ was obtained from the University of Thessaly in Greece (Table 1). The clean environment in lab rearing might have reduced the abundance of environmental fungi in the community of *L. serricorne* ‘chickpea’, leading to the increase of relative abundance of *Sy. buchneri*. In *L. serricorne* ‘greece’ the transfer between labs might have led to the high relative abundance of opportunistic environmental fungi like *Aspergillus*. Since we did not surface sterilise beetles before DNA extraction, fungal remains or spores from their surroundings could also appear in the community analyses. For example, in the sample of *L. serricorne* ‘forest’ we found mould fungi like *Alternaria* and *Cladosporium*.

In *St. paniceum* populations the *Symbiotaphrina* species could not be identified on species level based on the fungal community analysis. We included the obtained sequences of the corresponding ASVs in a phylogenetic analysis with the previously analysed *Symbiotaphrina* isolates. The ASVs clustered with the *Stegobium* ‘chili’ and *Stegobium* ‘cornflakes’ symbionts (Fig. 4). We observed almost no differences in the relative abundance of *Symbiotaphrina* between F0 and Fx in *St. paniceum* populations. We confirmed the identity of these YLS as *Sy. buchneri* and a novel *Symbiotaphrina* isolate using diagnostic primers in the individuals that were pooled for the fungal analysis.

The association of *Sy. buchneri* and “novel *Symbiotaphrina*” with *L. serricorne* and *St. paniceum* respectively was stable during lab rearing. The fungal community of lab reared beetles in F0 and Fx are very similar (Fig. 2) with the before mentioned exception of *L. serricorne* ‘greece’. Generation Fx of *L. serricorne* ‘greece’ clusters with both generations of *L. serricorne* ‘chickpea’ in the PCoA which can be evidence for a loss of unspecific opportunistic fungi. Since the YLS have an extracellular life phase during transmission from mother to offspring, the exchange with free-living *Symbiotaphrina* species might be possible in nature. Pant and Fraenkel^21^ described that sterilized larvae were able to acquire the YLS either by feeding on a diet supplemented with them or by feeding on a diet supplemented with faeces of normal insects. Experimentally, the YLS could be exchanged between host beetles^25^. However, the association of host beetle and YLS species seems to be rather stable in nature. The populations of *L. serricorne* and *St. paniceum* that we analysed here originated from different sources but were almost all associated with similar YLS strains although not always the previously reported ones. While this finding indicates more flexible associations than previously assumed, exchanges with other YLS species might still not happened too frequently in nature. Otherwise, an even higher YLS diversity between populations of the same beetle species would be expected.

Other anobiids are also associated with yeast-like symbionts^8^, for example symbionts of *Ernobius abietis*, *Ernobius mollis* and *Xestobium plumbeum* were isolated^13^ and described as *Candida karawaiewii*, *C. ernobii* and *C. xestobii* respectively^27^. However, in our fungal community analysis we also identified the novel *Symbiotaphrina* isolate in the *Ernobius* sample. A phylogenetic classification placed the YLS of other anobiids within the Saccharomycetales and clearly discriminated them from *Symbiotaphrina*^27^. The identification of *Symbiotaphrina* in this *Ernobius* sample suggests that other anobiids might also be associated with *Symbiotaphrina* species. However, the analysis of a single beetle sample is not representative and deeper research with more anobiid species is necessary. Since other anobiids feed on wood^8^, it is likely that the symbiosis between *L. serricorne* and *St. paniceum* with *Symbiotaphrina* arose from a common wood associated ancestor^10^.

In conclusion, our findings challenge the literature view of a strict and stable one on one symbiosis of anobiids and *Symbiotaphrina* with the commonly accepted association of *L. serricorne* – *Sy. kochii* and *St. paniceum* – *Sy. buchneri*. Most of the *L. serricorne* samples tested were associated with *Sy. buchneri* instead of *Sy. kochii*, while *St. paniceum* was consistently associated with a *Symbiotaphrina* strain that formed a distinct clade within our phylogenetic analysis, suggesting a more flexible association of anobiids and *Symbiotaphrina* YLS. However, the symbiosis in the lab-reared populations was stable with no exchanges happening between the beetle species, correspondingly populations of different origins were mostly associated with similar strains. Whether the novel strain represents a new *Symbiotaphrina* species with a unique metabolic repertoire or only a variant of the rRNA operon requires further genomic and phylogenetic analyses. Similarly, a wider screening is needed to reveal the precise dynamics of this association across habitats and the entire Ptinidae family.

Methods.

### Beetle strains

In this study several strains of *Lasioderma serricorne* and *Stegobium paniceum* were used partially obtained from long-term laboratory reared populations, partially collected from natural habitats and infested kitchen supplies and subsequent laboratory cultivation (Table 1). All populations from kitchen supplies were derived from independent households.

**Table 1:** Anobiid species, strains, their origin and status upon receiving the samples. F0: beetle samples directly frozen after receiving them in the lab “F0 generation”; fx: current generation of living beetles.

species	strain	origin	Sampling status
*Ernobius sp.*		Forest (Tegernheim, Germany)	Fresh frozen
*Lasioderma serricorne*	chickpea	chickpea flour (Mainz, Germany)	F0 fresh frozen, Fx 2-year lab culture
*Lasioderma serricorne*	chili	chili spice (Mainz, Germany)	2-year lab culture
*Lasioderma serricorne*	forest	Gonsenheim forest, (Mainz, Germany)	Fresh frozen
*Lasioderma serricorne*	Greece	Laboratory population, Greece (Professor Dr. Christos Athanassiou, University of Thessaly, Volos, Greece)	F0 fresh frozen, Fx 2-year lab culture
*Lasioderma serricorne*	JKI	Laboratory population, (Dr. Cornel Adler, Julius Kühn Institute Berlin, Germany)	Permanent lab culture
*Stegobium paniceum*	chili	chili spice (Rüsselsheim, Germany)	F0 fresh frozen, Fx 2-year lab culture
*Stegobium paniceum*	cornflakes	cornflakes (Mainz, Germany)	F0 fresh frozen, Fx 2-year lab culture
*Stegobium paniceum*	ginger	dried ginger root (Mainz, Germany)	Fresh frozen
*Stegobium paniceum*	JKI	Laboratory population (Dr. Cornel Adler, Julius Kühn Institute, Berlin, Germany)	Permanent lab culture

### Beetle rearing

*Stegobium paniceum* cultures ‘JKI’, ‘chili’ and ‘cornflakes’ as well as *L. serricorne* cultures ‘JKI’, ‘chickpea’, ‘chili’ and ‘greece’ were reared at 26 °C and 60–70% relative humidity and a 16/8 h light/dark cycle. Beetles were fed with equal parts by volume of oats, wheat bran and wheat germ with the addition of half a bread roll for *S. paniceum* respectively another volume of dried tobacco *L. serricorne*. Some specimens of each beetle population were frozen upon receiving them to conserve the information on their associated community before the attempt to establish lab rearing with the remaining specimen.

### Isolation of Yeast-like symbionts

Yeast-like symbionts were isolated from the currently reared beetle cultures *St. paniceum* ‘JKI’, ‘cornflakes’ & ‘chili’ as well as *L. serricorne* ‘JKI’, ‘greece’, ‘chickpea’ & ‘chili’ (Table 1). Entire individuals (adults or larvae) were surface sterilized in 70% ethanol for 1 min, twice washed in sterile cultivation medium for 30 seconds each. Subsequently, mycetomes were dissected, homogenized with a pipette tip in 100 µL of cultivation medium and spread on agar plates. Hansen’s broth or agar was prepared following Pant & Fraenkel^21^: 50 g/L glucose, 10 g/L peptone from soy, 3 g/L KH2PO4, 3 g/L MgSO4 and 15 g/L agar-agar (pH 6). Agar plates were supplemented with 15 mg/L tetracycline hydrochloride, 15 mg/L nalidixic acid and 25 mg/L chloramphenicol to suppress growth of bacteria. Growth of YLS colonies could be observed within three to twelve weeks, whereafter single colonies were transferred twice to agar plates described above and were afterwards maintained on Hansen’s agar or potato-dextrose agar containing 4 g/L potato infusion, 20 g/L glucose, 15 g/L agar-agar (pH 5.6) without the addition of antibacterial supplements. Culture plates were incubated within loosely closed, sterile plastic bags at 25 °C in the dark. Thereby we obtained seven strains of *Symbiotaphrina* in addition to two strains purchased from culture collections (Table 2).

**Table 2:** Isolated yeast- like symbiont strains and their origin.

YLS	species	strain	origin
*Symbiotaphrina buchneri*	*Symbiotaphrina buchneri*	NBRC 10,845	Institute for Fermentation, Osaka (ISO Japan); originally isolated at the National Institute of Sericulture and Entomological Science from gut cecum of *Stegobium paniceum*
*Symbiotaphrina kochii*	*Symbiotaphrina kochii*	CBS 250.77	Westerdijk Fungal Biodiversity Institute (CBS, Netherlands) originally isolated from *Lasioderma serricorne* (Jurzitza 1964)
*Stegobium* ‘chili’ symbiont	*Symbiotaphrina* sp.	chili-Sp	*Stegobium paniceum* ‘chili’
*Stegobium* ‘cornflakes’ symbiont	*Symbiotaphrina* sp.	cornflakes	*Stegobium paniceum* ‘cornflakes’
*Stegobium* ‘greece’ symbiont	*Symbiotaphrina* sp.	greece	*Stegobium paniceum* ‘greece’
*Lasioderma* ‘chickpea’ symbiont	*Symbiotaphrina* sp.	chickpea	*Lasioderma serricorne* ‘chickpea’
*Lasioderma* ‘chili’ symbiont	*Symbiotaphrina* sp.	chili-Ls	*Lasioderma serricorne* ‘chili’
*Stegobium* ‘JKI’ symbiont	*Symbiotaphrina* sp.	JKI-Sp	*Stegobium paniceum* ‘JKI’
*Lasioderma* ‘JKI’ symbiont	*Symbiotaphrina* sp.	JKI-Ls	*Lasioderma serricorne* ‘JKI’

### DNA- extraction

Beetle samples (Table 1) were either frozen (−20 °C) or freshly collected from lab reared cultures. They were homogenised individually using glass beads and a bead mill at 30 Hz for 1 min. YLS isolates were scraped off the agar plates and suspended in 300 µL Tissue and Cell Lysis Solution. DNA was extracted using the Epicenter MasterPure Complete DNA and RNA Purification kit (Lucigen, Wisconsin, USA) following the user’s instructions with the following modifications: 25 U zymolyase (Carl Roth, Germany) was added to the homogenised samples with Tissue and Cell Lysis Solution and samples were incubated at 35 °C for 30 min before proceeding with the protocol. DNA pellets were resuspended in 50 µL LOW TE buffer (10 mM Tris-HCl (pH 8.0) + 0.1 mM EDTA) and stored at −20 °C. DNA concentration and purity were measured using NanoDrop 1000 (Peqlab/Thermo Scientific, Wilmington, USA).

### Purification of DNA extracts

DNA extracts of bad quality were further purified with a phenol chloroform modification of the extraction kit to improve the removal of interfering compounds beyond the usual protein precipitation based on the addition of acetic acid. 250 µL Tissue and Cell Lysis Solution was added to the extracts. They were kept on ice for 3 to 5 min and 600 µL phenol/chloroform/isoamylalcohol was added. Samples were vigorously vortexed and incubated for 10 min at room temperature. After centrifuging at 6010 rcf for 5 min, the upper phase was carefully transferred into a new tube and the organic phase was discarded. 500 µL isopropanol were added to the samples, they were inverted for 30–40 times and stored at −20 °C for about 3 h. Samples were centrifuged for 10 min at 18,407 rcf and the supernatant was discarded. The pellet was washed with 200 µL of cold ethanol (70%) and centrifuged for 5 min at 18,407 rcf. The supernatant was discarded, the pellet was dried using a SpeedVac (Thermo Scientific, Waltham, MA, USA) and resuspended in 50 µL Low TE. Samples were stored at – 20 °C.

### Fungal community analysis

The fungal community of adult beetles of *L. serricorne* populations ‘chickpea’ and ‘greece’, as well as *St. paniceum* populations ‘chili’ and ‘cornflakes’ were analysed in two generations. Therefore, beetle samples were immediately frozen upon receiving the populations to conserve their microbial composition before rearing in the lab (“F0” generation). The remaining populations were reared and established as lab cultures. Out of each of the four beetle cultures ten living beetles were collected (“Fx” generation). In addition, a wild caught *Ernobius sp*, a *L. serricorne* ‘forest’ and a *St. paniceum* ‘ginger’ beetle and individuals from lab cultures *L. serricorne* ‘JKI’, ‘chili’ as well as *St. paniceum* ‘JKI’ were analysed. DNA was extracted individually for all samples (see above). For each of the described populations, 2–10 individual DNA extracts were pooled. The individual DNA concentrations were considered and an equal amount of DNA from each individual was used to contribute to a total DNA amount of circa 200 ng in each pool. Pools were sent for paired end Illumina MiSeq sequencing of fungal ITS region at StarSEQ (Mainz, Germany) using a primer pair that yields sequences of circa 300 bp length (forward 5’-CTTGGTCATTTAGAGGAAGTAA- 3’; reverse 5’ – GCTGCGTTCTTCATCGATGC- 3’).

Untrimmed Illumina MiSeq reads were obtained from StarSEQ. Primers at the 3’ ends were trimmed using cutadapt^28^ in miniconda. Reads were quality filtered with a threshold of 20 for quality scores and further trimmed using RStudio (2024.04.2). Forward and reverse reads were merged. Sequences smaller than 50 nucleotides were discarded and error rates were determined. Because dada2 package version 1.1. was used, dereplicating was necessary. Afterwards Amplicon Sequence Variants (ASVs) were inferred. Data from two sequencing runs were combined and the previously inferred ASVs were assigned to a taxonomy using the UNITE ITS fungal databank general release dynamics^29^. The FASTA sequences for all top 30 ASVs were extracted and manually checked in NCBI with BLASTn and UNITE. Wrong classifications were corrected and unclassified ASVs were determined if possible. Principal coordinate analysis based on Bray-Curtis dissimilarity was generated in RStudio (2024.04.2) using the “phyloseq” package. Statistical analysis of beta-diversity was conducted by permutational multivariate analysis of variance (PERMANOVA) in RStudio (2025.05.1) using “vegan” package.

### Phylogenetic placement of *Symbiotaphrina* isolates

The YLS isolated from *L. serricorne* and *St. paniceum* beetles (Table 2) were phylogenetically classified based on the partial rRNA operon. The partial rRNA operons of *St. paniceum* ‘chili’ symbiont, *St. paniceum* ‘cornflakes’ symbiont, *St. paniceum* ‘greece’ symbiont, *L. serricorne* ‘chickpea’ symbiont, *L. serricorne* ‘chili’ symbiont, *L. serricorne* ‘JKI’ symbiont (isolates H4 & C1) and lab cultures of *Sy. buchneri* (NBRC 10845) and *Sy. kochii* (CBS 250.77) were amplified using a LongAmp polymerase (New England Biolabs, Ipswich, USA) with different primer combinations (Supplementary Table 2). Amplification settings consisted of an initial denaturation at 94 °C for 3 minutes, 30 cycles of denaturation at 94 °C for 30 seconds, annealing at 48 °C for 60 seconds and elongation at 65 °C for 7 minutes, followed by final elongation step at 65 °C for 10 minutes. To amplify the partial rRNA operon from YLS from *Ernobius*,* L. serricorne* forest and *St. paniceum* ginger the beetle gDNA extracts were used. Additionally, parts of the rRNA operons of *St. paniceum* ‘JKI’ symbiont (isolates B4, E5, F3, G3, G5, H4, H5) and *L. serricorne* ‘JKI’ symbiont (isolate F3) were amplified with a peqlab Taq polymerase (VWR, Darmstadt, Germany) using multiple primer combinations (Supplementary Table 2): initial denaturation at 94 °C for 3 minutes, 30 cycles of denaturation at 94 °C for 30 seconds, annealing at 48 °C for 60 seconds and elongation at 72 °C for 120 seconds, final elongation step at 72 °C for 10 minutes. With this approach, we were able to obtain a minimum of one successful amplification per sample.

PCR- products were purified using the innuPREP PCRpure Kit (AnalyticJena, Jena, Germany) following the manufacturer’s instructions with the following modifications: first 100 µL binding buffer were added to the PCR products, samples were added to the spin filter and centrifuged for 2 min at 13,523 rcf. The PCR products were washed twice with 100 µL binding buffer firstly centrifuging for 2 min and secondly for 5 min at 13,523 rcf. The filter was dried open for 10–15 min. For elution of pure PCR-products 10 µL dH_2_0 were used.

Purified PCR products were Sanger sequenced by StarSEQ (Mainz, Germany). Since we used multiple primer combinations that resulted also in shorter fragment sequences of the rRNA operon, sequencing was done in forward and reverse direction for all positive PCRs. The sequences were edited using BioEdit 7.2.5^30^. As this resulted in multiple sequences per sample, individual alignments were created for each sample in Geneious prime (2023.0.3, Auckland, New Zealand) using MAFFT (v7.490)^31,32^. Afterwards, consensus sequences were obtained. If the individual sequences could not be aligned due to lacking overlap *Sy. buchneri* (DQ248313.1) or *Sy. kochii* (DQ248314) were used as a query to evaluate the size of the resulting gap for the consensus sequence. However, all sequences were derived from individual YLS colonies or initial ~ 8000 bp amplicons.

In addition to the consensus sequences generated in this study, sequences of closely related *Symbiotaphrina* species were obtained from NCBI, based on Baral et al.^16^. The final alignment was created in Geneious prime (2023.0.3, Auckland, New Zealand) using MAFFT (v7.490)^31,32^. Sequences of *Sy. buchneri* DQ248313.1 and *Sy. kochii* DQ248314 were cut to fit the general length of the other sequences.

From the final alignment containing 8050 bp, a Maximum likelihood tree was calculated using RAxML (8.2.11)^33^ in Geneious with GTR + G as substitution model and 10,000 bootstrapping replicates. A Bayesian inference tree was calculated using MrBayes (3.2.6)^34^ implemented in Geneious using the GTR + G substitution model and *Deltopyxis trianulispora* as the outgroup. MCMCs were set to 1,100,000 with 100,000 burn-in and 200 subsampling. Resulting trees were rooted to the outgroup *Deltopyxis trianulispora*.

### Diagnostic PCR

Based on the phylogenetic placement of *Symbiotaphrina* isolates and the underlying alignment, diagnostic primers for distinction of *Symbiotaphrina* isolate groups were designed.

The first group, containing the sequences of *Stegobium* ‘greece’ symbiont, *Lasioderma* ‘chickpea’ symbiont, *Lasioderma* ‘JKI’ symbiont and *Stegobium* ‘JKI’ symbiont clustered with *Sy. buchneri* type strains. The second group containing *Stegobium* ‘chili’ symbiont and *Stegobium* ‘cornflakes’ symbiont clustered as a sister group to the fist group. One forward primer was designed for each group: Sym_buch_classic (5’ –GCCGATGTTCGTTCTCG – 3’) targeting the ITS region of *Sy. buchneri* type strains, *Stegobium* ‘greece’ symbiont and *Lasioderma* ‘chickpea’ symbiont and Sym_novel (5’ – CGTTGTCTGCTCTCACGAG – 3’) targeting the ITS region of *Stegobium* ‘chili’ symbiont and *Stegobium* ‘cornflakes’ symbiont. The online tool Primer3 (v.1.4.1)^35–37^ was used to optimise primer length and to avoid self-complementarity. Paired with ITS4 (Supplementary Table 2) as a reverse primer, they should yield PCR-products of circa 500 bp length. PCR conditions were optimized with a gradient of annealing temperatures. The best conditions for Sym_buch_classic were an initial denaturation at 94 °C for 3 min, 40 cycles of denaturation at 94 °C for 30 s, annealing at 57,5 °C for 60 s and elongation at 72 °C for 120 s, and final elongation at 72 °C for 10 min. The best conditions for Sym_novel were an initial denaturation at 94 °C for 3 min, 40 cycles of denaturation at 94 °C for 30 s, annealing at 63 °C for 60 s and elongation at 72 °C for 120 s, and final elongation at 72 °C for 10 min.

To distinguish both *Symbiotaphrina* sp. strain groups in the samples that were used in the fungal community analysis, PCRs with both primers were run with the individual DNA extracts of *L. serricorne* ‘chickpea’ F0 & Fx, *L. serricorne* ‘greece’ F0 & Fx, *L. serricorne* ‘JKI’, *L. serricorne* ‘forest’, *L. serricorne* ‘chili’, *St. paniceum* ‘chili’ F0 & Fx, *St. paniceum* ‘cornflakes’ F0 & Fx, *St. paniceum* ‘JKI’, *St. paniceum* ‘ginger’ and *Ernobius*.

Since the *L. serricorne* ‘chili’ symbiont clustered with *Sy. kochii* and PCRs using Sym_buch_classic and Sym_novel were negative, a diagnostic PCR for *Sy. kochii* was performed. The primer pair S_kochii_fwd2 (5’ –GCTCAGCCGTGGTTCTCC– 3’) and S_kochii_rev2 (5’ –CCGAAGAGAGCTACATTCCC– 3’) targeting the 28 S region of *Sy. kochii* was used with an initial denaturation at 95 °C for 3 min, 40 cycles of denaturation at 95 °C for 30 s, annealing at 62 °C for 30 s and elongation at 72 °C for 30 s, and final elongation at 72 °C for 3 min.

Amplicons were analysed on an 1,6% agarose gel running for 30–40 min at 130 V using 3 µL PCR product mixed with 2 µL loading buffer.

## Supplementary Information

Below is the link to the electronic supplementary material.

Supplementary Material 1

## Data Availability

Sequence data is deposited on under Bioproject PRJNA1210556, Genbank PX406542-PX406559 and Edmond [38].
