# Supplementary material for: The stored product beetles Lasioderma serricorne and Stegobium paniceum are associated with a flexible and hidden diversity of Symbiotaphrina symbionts
Source: Sci Rep. 2026 Jan 8;16:1294. doi: 10.1038/s41598-025-34676-y (PMC12791133; doi:10.1038/s41598-025-34676-y)
Supplement: Supplementary file 1 — Supplementary Material 1 [file 41598_2025_34676_MOESM1_ESM.docx]

Supplementary information

The stored product beetles *Lasioderma serricorne* and *Stegobium paniceum* are associated with a flexible and hidden diversity of *Symbiotaphrina* symbionts

Nick, Alina; Schellenberg, Rene; Athanassiou, Christos G., Adler, Cornel; Engl, Tobias^*^

* corresponding author: [tengl@ice.mpg.de](mailto:tengl@ice.mpg.de)


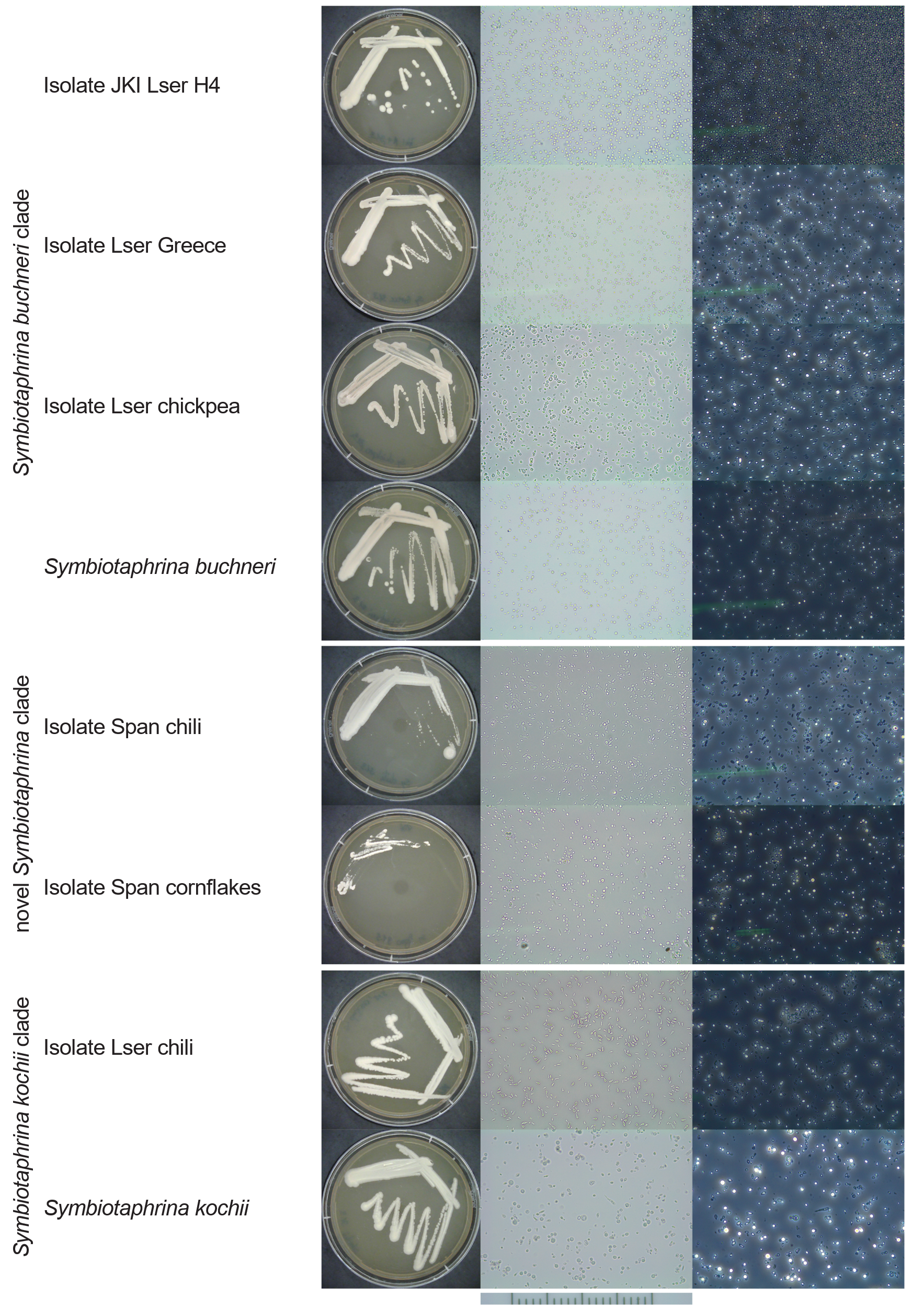


Supplementary Figure 1: Colony and cell morphology of *Symbiotaphrina* cultures used in this study. Diameter of the petri dishes is 9cm. Scale bar for bright field and phase contrast images is in total 200µm; smallest sub division is 10µm.


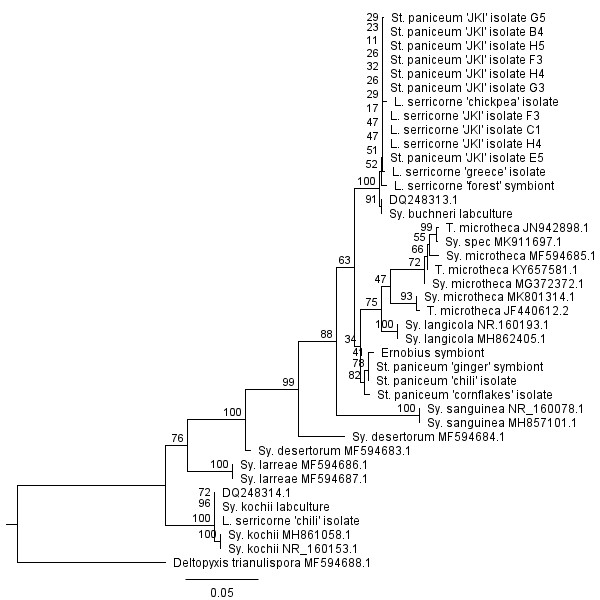


Supplementary Figure 2: Maximum likelihood phylogeny of Symbiotaphrina isolates and uncultured symbionts based on partial rRNA gene. RAxML, bootstrap values are given on branches


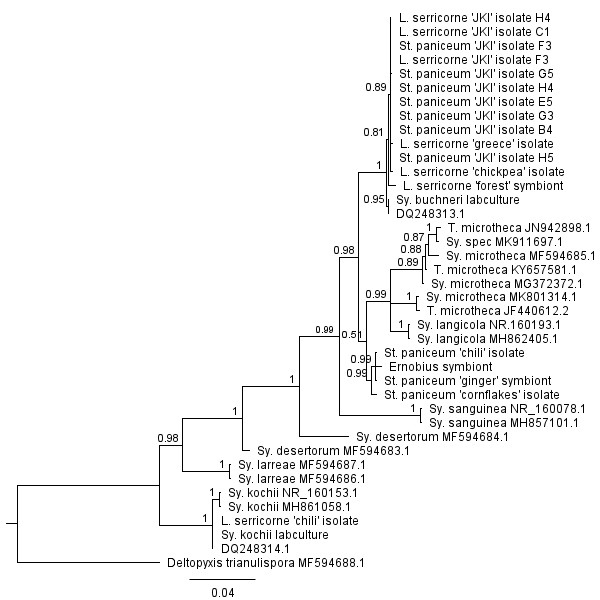


Supplementary Figure 3: Bayesian phylogeny of Symbiotaphrina isolates and uncultured symbionts based on partial rRNA gene. MrBayes, posterior probabilities are given on branches


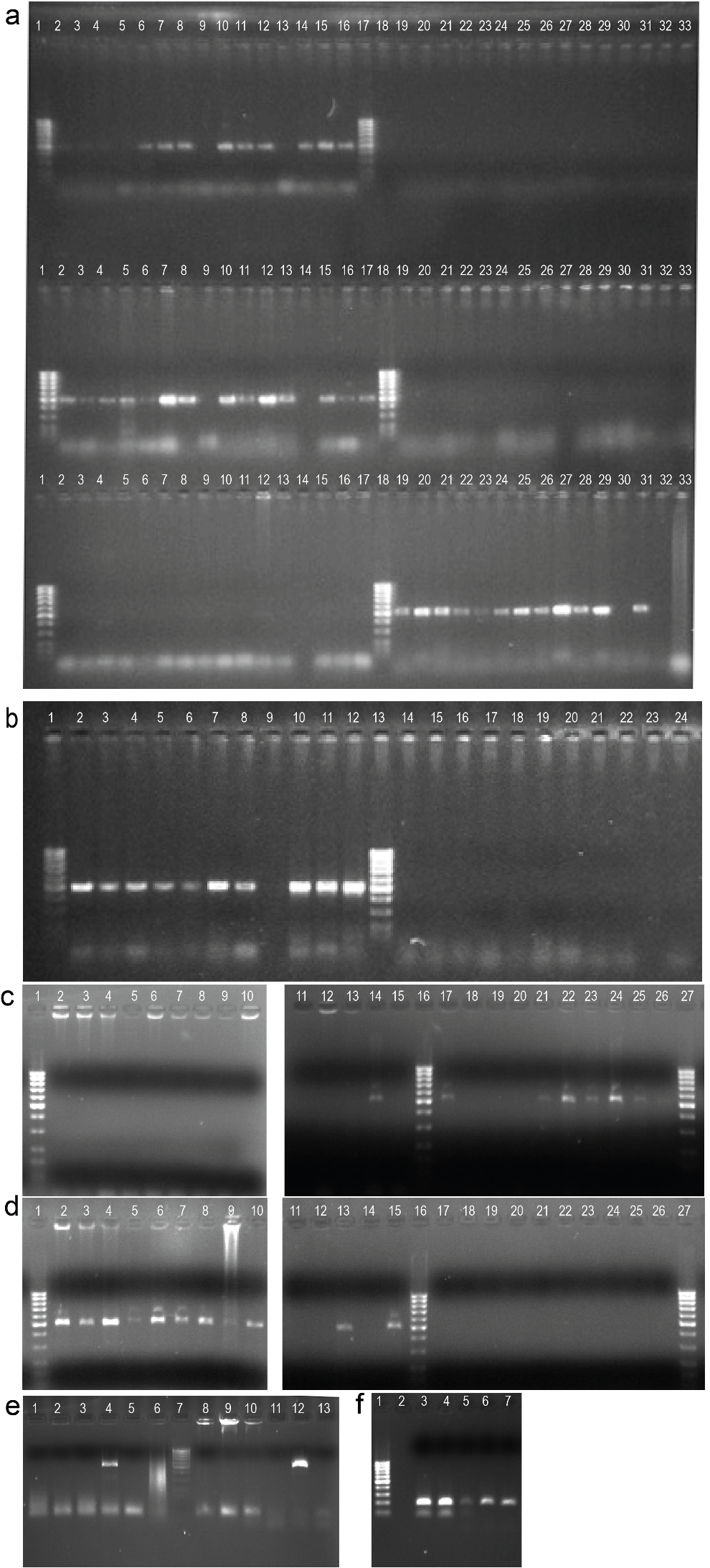


Supplementary Figure 4: Agarose gels of PCR products of diagnostic PCR of samples used in the fungal community analysis using diagnostic primers Sym_buch_classic and Sym_buch_novel. Details on sample order in Supplementary Table S2

Supplementary Table 1: Details on sample order in the gels in Supplementary Figure S3.

| **gel panel** | **row** | **slot** | **sample** | **primer** |
| --- | --- | --- | --- | --- |
| a | top | 1 | 100 bp ladder |  |
|  |  | 2-6 | L. serricorne chickpea F0 1-5 | Sym_buch_classic + ITS4 |
|  |  | 7-16 | L. serricorne chickpea Fx 1- 10 |  |
|  |  | 17 | 100 bp ladder |  |
|  |  | 19-23 | L. serricorne chickpea F0 1-5 | Sym_buch_novel+ ITS4 |
|  |  | 24-33 | L. serricorne chickpea Fx 1-10 |  |
|  | middle | 1 | 100 bp ladder |  |
|  |  | 2-6 | L. serricorne greece F0 1-5 | Sym_buch_classic+ ITS4 |
|  |  | 7-13 | L. serricorne greece Fx 1-7 |  |
|  |  | 15- 17 | L. serricorne greece Fx 8- 10 |  |
|  |  | 18 | 100 bp ladder |  |
|  |  | 19- 23 | L. serricorne greece F0 1- 5 | Sym_buch_novel+ ITS4 |
|  |  | 24- 33 | L. serricorne greece Fx 1- 10 |  |
|  | bottom | 1 | 100 bp ladder |  |
|  |  | 2- 6 | St. paniceum chili F0 1- 5 | Sym_buch_classic+ ITS4 |
|  |  | 7- 13 | St. Paniceum chili Fx 1- 7 |  |
|  |  | 15- 17 | St. Paniceum chili Fx 8-10 |  |
|  |  | 18 | 100 bp ladder |  |
|  |  | 19- 23 | St. paniceum chili F0 1- 5 | Sym_buch_novel+ ITS4 |
|  |  | 24- 33 | St. Paniceum chili Fx 1- 10 |  |
| b |  | 1 | 100 bp ladder |  |
|  |  | 2- 8 | L. serricorne JKI 3- 9 | Sym_buch_classic+ ITS4 |
|  |  | 10- 12 | L. serricorne JKI 10 -12 |  |
|  |  | 13 | 100 bp ladder |  |
|  |  | 14- 22 | L. serricorne JKI 3 -11 | Sym_buch_novel+ ITS4 |
|  |  | 24 | L. serricorne JKI 12 |  |
| c |  | 1 | 100 bp ladder |  |
|  |  | 2-3 | St. paniceum cornflakes F0 1-2 | Sym_buch_classic+ ITS4 |
|  |  | 4-13 | St. paniceum cornflakes Fx 1- 10 |  |
|  |  | 14 | L. serricorne forest |  |
|  |  | 15 | Ernobius |  |
|  |  | 16 | 100 bp ladder |  |
|  |  | 17-26 | St. paniceum JKI 2- 10, 12 | Sym_buch_classic+ ITS4 |
|  |  | 27 | 100 bp ladder |  |
| d |  | 1 | 100 bp ladder |  |
|  |  | 2-3 | St. paniceum cornflakes F0 1-2 | Sym_buch_novel+ ITS4 |
|  |  | 4-13 | St. paniceum cornflakes Fx 1- 10 |  |
|  |  | 14 | L. serricorne forest |  |
|  |  | 15 | Ernobius |  |
|  |  | 16 | 100 bp ladder |  |
|  |  | 17-26 | St. paniceum JKI 2- 10, 12 | Sym_buch_novel+ ITS4 |
|  |  | 27 | 100 bp ladder |  |
| e |  | 1-3 | L. ser chili 1-3 | Sym_buch_novel+ ITS4 |
|  |  | 4 | S. pan ginger |  |
|  |  | 5 | Negative control |  |
|  |  | 6 | Positive control Symbiotaphrina isolate chili |  |
|  |  | 7 | 100 bp ladder |  |
|  |  | 8-10 | L. ser chili 1-3 | Sym_buch_classic+ ITS4 |
|  |  | 11 | S. pan ginger |  |
|  |  | 12 | Positive control *Sy. buchneri* DNA |  |
|  |  | 13 | Negative control |  |
| f |  | 1 | 100 bp ladder | S_kochii_fwd2 + S_kochii_rev2 |
|  |  | 3-5 | L. serricorne ‘chili’ 1-3 beetle extract |  |
|  |  | 6 | L. serricorne ‘chili’ yeast isolate extract |  |
|  |  | 7 | Positive control Sy. kochii DNA |  |

Supplementary Table 2: Primer for amplification and sequencing of fungal rRNA operon.

| primer | direction | Sequence 5‘ -> 3‘ | target | reference |
| --- | --- | --- | --- | --- |
| LR5 | reverse | ATCCTGAGGGAAACTTC | Fungal 28 S rRNA  Fungal 28 S rRNA | 1 |
| Fungi_LS1 | forward | TACCCGCTGAACTTAAG |  | 2,3 |
| ITS1 | forward | TCCGTAGGTGAACCTGCGG | ITS region between 18 S and 28 S rRNA of fungi | 4 |
| ITS4 | reverse | TCCTCCGCTTATTGATATGC |  |  |
| ITS5 | forward | GGAAGTAAAAGTCGTAACAGG |  |  |
| S_buchneri_fwd1 | forward | CTGCAGTTGATCAACCGGT | *Sy. buchneri* 28 S | This study |
| S_buchneri_fwd2 | forward | CGGTGCACTCTTCTGCAGA |  |  |
| S_buchneri_rev2 | reverse | GCCTTTATCCAACCACCCAAACT |  |  |
| S_kochii_rev1 | reverse | CCCGACCTTTATCCAGCCG | *Sy. kochii* 28S |  |
| S_kochii_rev2 | reverse | CCGAAGAGAGCTACATTCCC |  |  |
| S_kochii_fwd2 | forward | GCTCAGCCGTGGTTCTCC |  |  |
| Sym_buch_classic | forward | GCCGATGTTCGTTCTCG | ITS region of *Sy. buchneri* type strains, *Stegobium* ‘greece’ symbiont & *Lasioderma* ‘chickpea’ symbiont |  |
| Sym_novel | forward | CGTTGTCTGCTCTCACGAG | ITS region of *Stegobium* ‘chili’ symbiont & *Stegobium* ‘cornflakes’ symbiont |  |

References

1 Vilgalys, R. & Hester, M. Rapid genetic identification and mapping of enzymatically amplified ribosomal DNA from several Cryptococcus species. *Journal of Bacteriology* **172**, 4238-4246 (1990).

2 Hausner, G., Reid, J. & Klassen, G., R. On the phylogeny of Ophiostoma, Ceratocystis s.s., and Microascus, and relationships within Ophiostoma based on partial ribosomal DNA sequences. *Canadian Journa of Botany* **71**, 1249-1265 (1993).

3 Gibson, C. M. & Hunter, M. S. Inherited fungal and bacterial endosymbionts of a parasitic wasp and its cockroach host. *Microb Ecol* **57**, 542-549 (2009). <https://doi.org/10.1007/s00248-008-9436-1>

4 White, T., J, Burns, T., D, Lee, S., B & Taylor, J., W. in *PCR - Protocols and Applications - A Laboratory Manual* 315-322 (Academic Press, 1990).
